# Biosynthetic Potential of Bioactive Streptomycetes Isolated From Arid Region of the Thar Desert, Rajasthan (India)

**DOI:** 10.3389/fmicb.2018.00687

**Published:** 2018-04-18

**Authors:** Meeta Masand, Kunjukrishnan Kamalakshi Sivakala, Ekta Menghani, Thangathurai Thinesh, Rangasamy Anandham, Gaurav Sharma, Natesan Sivakumar, Solomon R. D. Jebakumar, Polpass Arul Jose

**Affiliations:** ^1^School of Life Sciences, Suresh Gyan Vihar University, Jaipur, India; ^2^Department of Molecular Microbiology, School of Biotechnology, Madurai Kamaraj University, Madurai, India; ^3^Department of Biotechnology, School of Sciences, JECRC University, Jaipur, India; ^4^Department of Microbiology, School of Life Sciences, Pondicherry University, Puducherry, India; ^5^Department of Agricultural Microbiology, Agricultural College and Research Institute, Tamil Nadu Agricultural University, Madurai, India

**Keywords:** arid, desert, *Streptomyces*, metabolomics, secondary metabolome, antimicrobial activity

## Abstract

Acquisition of Actinobacteria, especially *Streptomyces* from previously underexplored habitats and the exploration of their biosynthetic potential have gained much attention in the rejuvenated antibiotics search programs. Herein, we isolated some *Streptomyces* strains, from an arid region of the Great Indian Thar Desert, which possess an ability to produce novel bioactive compounds. Twenty-one morphologically distinctive strains differing in their aerial and substrate mycelium were isolated by employing a stamping method. Among them, 12 strains were identified by a two-level antimicrobial screening method, exerting antimicrobial effects against a panel of indicator strains including methicillin-resistant *Staphylococcus aureus* and vancomycin-resistant *Enterococcus* species. Based on their potent antimicrobial activity, four isolates were further explored by 16S rRNA gene-based identification, genetic screening, and metabolomic analysis; and it was found that these strains belong to the genus *Streptomyces*. The selected strains were found to have polyketide synthase and non-ribosomal peptide synthetase systems. In addition, extracellular metabolomic screening revealed that the isolates produced analogs of doxorubicinol, pyrromycin, erythromycin, and 6-13 other putative novel metabolites. These results demonstrate the significance of *Streptomyces* inhabiting the arid region of Thar Desert, suggesting that similar arid environments can be considered as the reservoirs of novel *Streptomyces* strains that could have biotechnological significance.

## Introduction

The escalating levels of antibiotic resistance in pathogenic microorganisms clearly dictate the need for identifying new bioactive compounds for novel drug discovery. Microorganisms, especially Actinobacteria, have been isolated from diverse environments and have most frequently been explored as a novel source for the production of bioactive compounds (Peraud et al., 2009; Poulsen et al., 2011; Subramani and Aalbersberg, 2012; Jose et al., 2013; Jose and Jebakumar, 2014; Sun et al., 2015; Singh et al., 2016). In the past 75 years, the Actinobacteria have exerted a major impact on the discovery of antibiotics and several other drugs (Baltz, 2007; Procópio et al., 2012), and continue as one of the most important sources of chemical diversity. Consequently, it makes good sense to isolate and systematically identify the potent candidates affiliated to Actinobacteria in the search for new bioactive compounds.

Recently, the search for bioactive metabolites has intensified worldwide, aided by the advancements in next generation sequencing technologies (Gomez-Escribano et al., 2016), spectroscopic methods (Forner et al., 2013; Derewacz et al., 2015; Floros et al., 2016), and bulk data processing platforms (Floros et al., 2016). There is notable rejuvenation in secondary metabolites search from *Streptomyces* (Gomez-Escribano et al., 2016), attested by increasing number of genome announcements and guided discoveries (Bachmann et al., 2014; Ju et al., 2015; Gomez-Escribano et al., 2016). Moreover, there are a number of reports on novel isolates with biosynthetic potential from novel sources explored by metabolomic and genomic screening methods (Guo et al., 2015; Sengupta et al., 2015).

The Actinobacteria associated with unexplored and underexplored habitats are considered as a novel source of biologically active metabolites (Guo et al., 2015; Sengupta et al., 2015). In this context, deserts are underexplored environments with limited reports on their microbiota endowed with the potential to produce novel bio-active metabolites (Takahashi et al., 1996; Hozzein et al., 2008; Okoro et al., 2009). Moreover, the desert environments (Mohammadipanah and Wink, 2016) are relatively less explored as source of novel Actinobacteria when compared to endosphere of plants (Masand et al., 2015), marine sediments (Jose and Jha, 2017), and marine animals (Mahmoud and Kalendar, 2016; Dhakal et al., 2017). A high diversity and suggested endemicity of culturable Actinobacteria have recently been found in an extremely oligotrophic desert oasis (Arocha-Garza et al., 2017).

The Great Indian Thar Desert is the ninth largest subtropical desert, which comprises of a range of arid to subhumid climatic conditions. Reports on the exploration and/or exploitation of Actinobacteria in this region are very limited, with the exception of a recent finding which claimed the recovery of 13 bioactive Actinobacteria from the Jaisalmer and Jodhpur regions of Thar Desert (Tiwari et al., 2015). This study was carried out in Bikaner, which is located right in the middle of the Thar Desert. While there is no previous report, our current focus is based on the modern metabolomics based assessment for novel *Streptomyces* with potential for producing bioactive metabolites. This has led to the isolation of potentially active strains that produce metabolites against pathogenic bacterial strains. We describe the isolation, molecular identification, antimicrobial potential, and secondary metabolites from these isolates.

## Materials and Methods

### Location and Collection of Soil Samples

**FIGURE 1:**
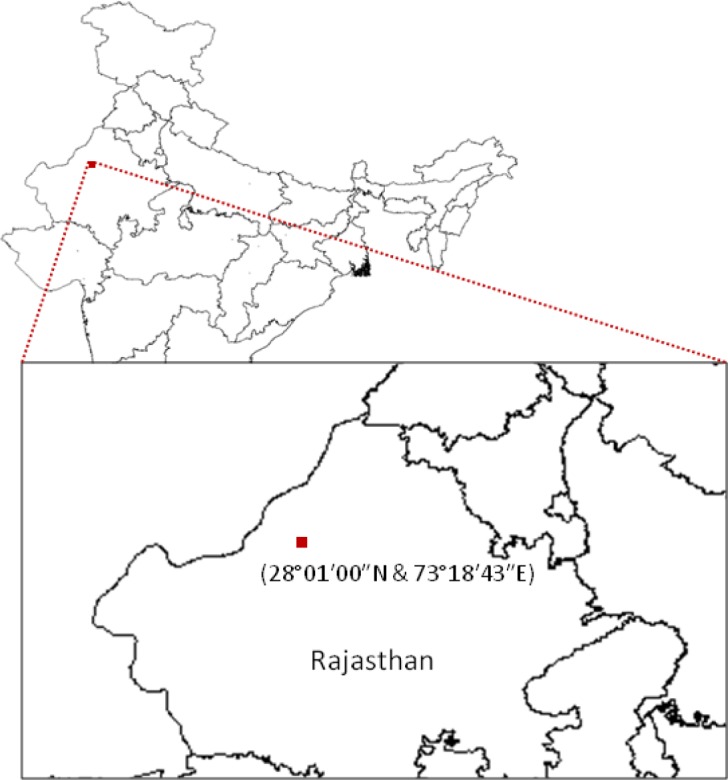
Sampling site. The map shows the sample collection site in the Thar Desert, Rajasthan (India).

Arid soil samples were collected from Bikaner, located in the Great Indian Thar Desert (**Figure 1**). Bikaner is situated between 28°01′00′′N and 73°18′43′′E, and has a hot arid climate. A total of eight soil samples were collected within a 128 m^2^ plot at a depth of 10-15 cm from the upper surface of the topsoil and stored at 4°C in labeled sterile bags before transporting to our laboratory for further processing.

### Isolation Media

Three different isolation media were employed: starch casein agar (10.0 g soluble starch, 0.3 g casein, 2.0 g KNO_3_, 2.0 g NaCl, 2.0 g K_2_HPO_4_, 0.05 g MgSO_4_.7H_2_O, 0.02 g CaCO_3_, 0.01 g FeSO_4_.7H_2_O, 20.0 g agar in 1,000 mL distilled water; pH 7.0), Actinobacteria isolation agar (2.0 g sodium caseinate, 0.1 g asparagine, 4.0 g sodium propionate, 0.5 g K_2_HPO_4_, 0.1 g MgSO_4_.7H_2_O, 0.001 g FeSO_4_.7H_2_O and 20.0 g agar in 1,000 mL distilled water; pH 7.0) and modified MM agar (1.0 g glucose, 0.5 g yeast extract, 1.0 g K_2_HPO_4_, 0.5 g MgSO_4_⋅7H_2_O, and 0.5 g NaCl, and 20 g agar). All the three media were supplemented with antibiotics, cycloheximide (100 μg mL^-1^), and nalidixic acid (25 μg mL^-1^) to reduce fungal growth and contamination by fast growing bacteria.

### Sample Processing and Isolation of the Actinobacteria

All the soil samples were dried in a laminar flow hood for 24 h. The samples were then processed by using the heat-shock-and-dilution and plate stamping methods (Mincer et al., 2002; Jose and Jebakumar, 2013) with a minor modification, and inoculated onto the agar media. In heat-shock-and-dilution method, sterile water was used in the place of seawater. The plates were incubated for 2–10 weeks at 28–32°C and observed for the appearance of Actinobacteria-like colonies. Well-separated Actinobacteria-like colonies were removed from the original isolation plates and sub-cultured on modified ISP 4 medium (Jose and Jebakumar, 2013) to recover pure cultures with uniform colony morphology. Pure cultures of the isolates were again streaked on modified ISP4 medium and incubated for 10 days at 30°C. Aerial mycelium, substrate mycelium, pigmentation and sporulation were observed after the incubation period, and unique isolates were selected for further studies.

### Screening of Isolates for Antimicrobial Activity

Isolates collected from the semi-arid soil samples were screened for antibacterial activity by cross streak (Lemos et al., 1985) and agar-plug methods (Jose and Jebakumar, 2013) against four bacteria and a yeast strain. In the cross streak method, the isolates were streaked on starch casein agar and modified ISP4 agar medium, and incubated at 29 ± 2°C for 7 days to obtain a copious ribbon-like growth. Then, overnight cultures of three clinical pathogens [methicillin-resistant *Staphylococcus aureus* (MRSA), vancomycin-resistant *Enterococcus* (VRE), and *Candida albicans*], and two type strains (*Pseudomonas aeruginosa* ATCC 10145, *Escherichia coli* ATCC 3739) were cross streaked at a 90° angle to actinobacterial cultures. Subsequently, plates were incubated at 29 ± 2°C for 24 h, and the zone of inhibition was observed.

In the agar plug method, the isolates were initially inoculated in the agar medium described above and incubated until sufficient growth was achieved. Then, agar plugs of 6 mm in diameter were cut from the 10-day-old agar plates and plugged into wells, bored using a sterile cork borer (diameter of 6 mm), in the Mueller–Hinton agar plates, which were seeded with bacteria and yeast. The agar plugged plates were incubated at 37°C for 24 h and observed for the zone of inhibition around the inserted agar plugs. Clinical pathogens used in this study were obtained from Kovai Medical Centre and Hospital (KMCH, Coimbatore, India).

### 16S rRNA Gene Amplification and Phylogenetic Analysis

Four potential isolates were selected for 16S rRNA gene sequencing and phylogenetic analysis. The strains were cultured in 50 mL of trypticase soy broth, shaken at 180 rpm at 29 ± 2°C for 7 days, and the resulting mycelia biomass was pelleted by centrifugation. Genomic DNA was extracted using HiPurA *Streptomyces* genomic DNA purification kit (HiMedia, India), according to the manufacturer’s protocol. The 16S rRNA gene was amplified using universal 27F (5′-AGA GTTTGATCCTGGCTCA-3′) and 1492R (5′-ACGGCTACCTTGTTACGACT-3′) primers with the following thermal cycling conditions: initial denaturation at 95°C for 5 min followed by 32 cycles at 95°C for 30 s, 55°C for 90 s and 72°C for 120 s, followed by a final extension at 72°C for 5 min. The reactions were performed in MyCycler (Bio-Rad, United States) and the amplified products were examined by 1% agarose gel electrophoresis. The PCR products were purified and sequenced by Applied Biosystems 3730XL DNA analyzer. The forward and reverse 16S rRNA gene sequences, obtained from each strain, were assembled and analyzed using Basic Local Alignment Search Tool (Altschul et al., 1990). To identify the isolates, the sequences were uploaded to EzTaxon server (Kim et al., 2012) and compared with 16S rRNA gene sequence of the type strains. The Ribosomal Database Project online tool (Cole et al., 2014) was used to align the sequences with the related 16S rRNA gene sequences from GenBank and downloaded in the FASTA format. The sequence file was imported into MEGA5 (Tamura et al., 2011) and a neighbor-joining phylogenetic tree was constructed with 1,000 bootstrap replicates.

### Extraction of Antibacterial Compounds

Selected antagonistic actinobacterial strains SAS02, SAS09, SAS13, and SAS15 were inoculated from frozen stocks into 50 mL of modified ISP4 medium and shaken at 180 rpm and 29 ± 2°C for 3 days. The cultures were then transferred to 1 L of the same medium and incubated at the same condition for 7 days. After incubation, the cultures were filtered and the cell-free filtrate was extracted with an equal volume of ethyl acetate which is often employed for secondary metabolites extraction (Becerril-Espinosa et al., 2013; Cheng et al., 2015; Wu et al., 2016). The organic layer was separated, concentrated under vacuum, and dissolved in methanol until HR-LCMS analysis.

### PCR-Based Screening for Biosynthetic Systems

Ketosynthase (KS) and adenylation domain fragments of polyketide synthase (PKS) and non-ribosomal peptide synthetase (NRPS) systems, respectively, were PCR amplified using 20–50 ng of genomic DNA. The degenerate primers used in the study are given in **Table 1**. PCR conditions were as follows: an initial denaturation at 95°C for 5 min, followed by 31 cycles of 30 s at 95°C, 1 min at 58°C (KS) or 61°C (NRPS), and 1 min at 72°C, and a final extension at 72°C for 7 min. The tubes without template DNA were used as negative control and the tubes with DNA of previously characterized strain *Streptomyces* sp. JAJ06 (Jose et al., 2011) bearing PKS and NRPS systems were considered as positive controls. PCR products were checked by agarose gel (1%, w/v) electrophoresis.

**Table 1:** Primers used in PCR-based screening of isolates for PKS and NRPS biosynthetic systems.

Gene	Primer (5′ to 3′)	Reference
PKS-ketosynthase domain	TSGCSTGCTTGGAYGCSATC (F) TGGAANCCGCCGAABCCTCT (R)	Metsa-Ketela et al., 1999
NRPS-adenylation domain	GCSTACSYSATSTACACSTCSGG (F) SASGTCVCCSGTSCGGTAS (R)	Ayuso-Sacido and Genilloud, 2005

### HR-LCMS Analysis

The crude extracts were subjected to HR-LCMS analysis in an Infinity Nano HPLC-Chip cube system coupled with iFunnel MS-Q-TOFs (Agilent Technologies, United States). The sample injection volume was 5 μL. The compound separation was performed at a flow rate of 0.3 mL/min under a gradient program. Mobile phase A was HPLC grade water containing 0.1% (v/v) formic acid and mobile phase B was HPLC grade acetonitrile supplemented with 10% (v/v) water and 0.1% (v/v) formic acid. The separation was performed in gradient mode, using 5–95% of mobile phase B in mobile phase A over 20 min. For MS, dual Agilent Jet Stream ESI source was used and data were acquired in both positive and negative modes. Spectral data were analyzed using Agilent Mass Hunter Qualitative Analysis software (version B.05.00; Agilent Technologies) and compared with known compounds based on similar molecular features. HR-LCMS analysis was performed at the Sophisticated Analytical Instrumentation Facility (SAIF), Indian Institute of Technology, Mumbai, India.

## Results

### Isolation of Actinobacteria

**FIGURE 2:**
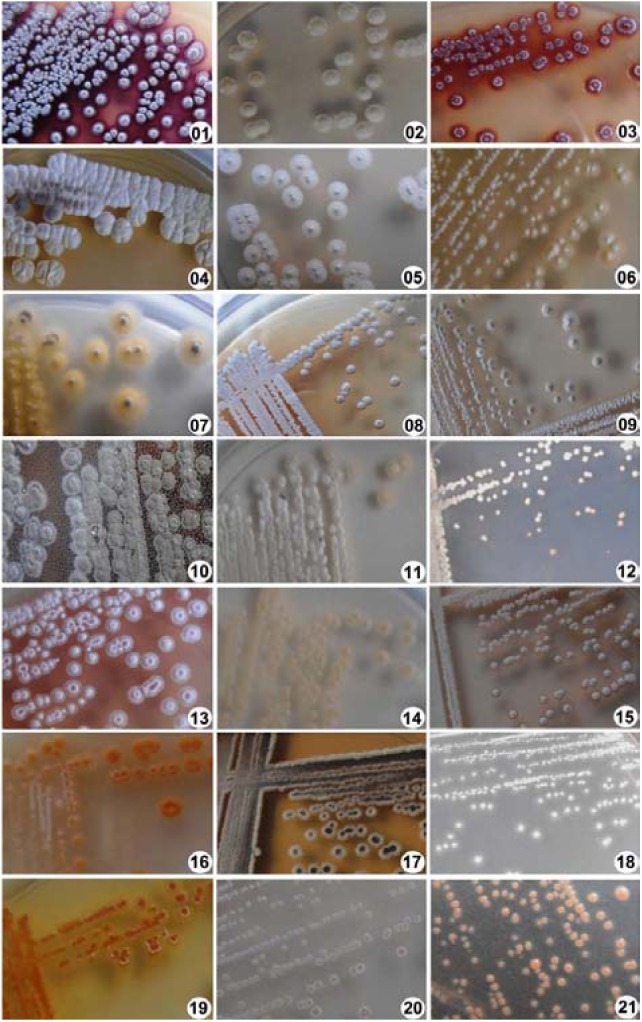
Morphological characterization of actinobacterial isolates. Colony morphology of different actinobacterial isolates derived from semi-arid soil samples was photographed after growing on modified ISP4 media for 10 days.

A total of 21 morphologically unique Actinobacteria were isolated from the eight soil samples collected from the Thar Desert. The pure culture of the isolates was obtained by a series of repeated streaking on an agar medium. The isolates that showed distinct colony morphology (**Figure 2**), indicative of the order Actinomycetales, were taken for further analysis. Among the two different pre-treatment techniques employed, plate stamping resulted in the isolation of 16 (76%) strains. Heat-shock-dilution method resulted in isolation of relatively less number of (5, 24%) strains. Morphological characteristics of the 21 isolates are summarized in **Table 2**.

**Table 2:** Morphological characteristics of actinobacterial isolates acquired from the desert soils.

Isolates	Aerial mycelium	Substrate mycelium	Pigmentation
SAS01	Dark pink with gray spores	pink	Dark pink
SAS02	Creamy white with white spores	Light yellow	No
SAS03	Red with gray spores	Red	Red
SAS04	White	White	Yellow
SAS05	White	White	No
SAS06	White	Yellow	Pale yellow
SAS07	Yellow with dark gray spores	Yellow	No
SAS08	White	Yellowish white	Yellowish orange
SAS09	Gray	white	No
SAS10	Gray	Gray	Brown
SAS11	White	Pale yellow	No
SAS12	White pinpoint colonies	White	No
SAS13	White	Light pink	Light pink
SAS14	Yellow	Yellow	No
SAS15	Gray	Light pink	Light brownish
SAS16	Orange with white spores	Orange	Pale orange
SAS17	White and dark blue	Dark blue	No
SAS18	White	White	No
SAS19	Orange	Orange	Yellow
SAS20	White	White	No
SAS21	Pale pink	Pale pink	No

### Screening for Antimicrobial Activity

We screened the different isolates at two levels with two different production media against both the clinical pathogens (clinical isolates) and ATCC type strains. Notably, in the cross-streak method, where the antimicrobial activity of the isolates was tested against one yeast and five different bacterial strains, a total of 12 (57%) actinobacterial isolates were found to produce bioactive metabolites, in either or both SCA and M-ISP4, against at least one of the test strains. The antimicrobial metabolite producers included the following strains: SAS01–SAS06, SAS08, SAS09, SAS11, SAS13, SAS15, and SAS19.

**FIGURE 3:**
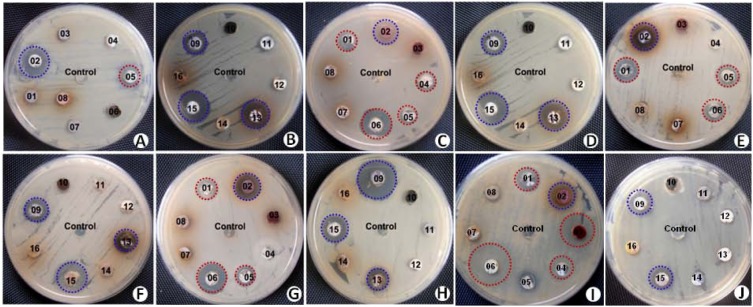
Screening of actinobacterial isolates for antimicrobial activity against series of test strains by agar-plug method. Antimicrobial assay plates showing antimicrobial activity of selected strains (shaded with blue color) against *Pseudomonas aeruginosa* ATCC 10145 **(A,B)**, *Escherichia coli* ATCC 3739 **(C,D)**, vancomycin-resistant *Enterococcus* **(E,F)**, methicillin-resistant *Staphylococcus aureus* **(G,H)**, and *Candida albicans* **(I,J)**.

Results of secondary screening for antimicrobial activity were presented as the diameter of inhibition zone against the test strains (**Table 3**). Out of the 12 antimicrobial isolates identified, nine (75%) exhibited considerable inhibitory activity against both Gram-negative and Gram-positive bacteria. The remaining three (25%) isolates exhibited antibacterial activity against only Gram-positive bacteria. In the case of antifungal activity against *Candida albicans*, except for SAS08 and SA11, all the isolates showed considerable growth inhibition. Based on the broad-spectrum activity exhibited, strains SAS02, SAS09, SAS13, and SAS15 (**Figure 3**) were selected for further analysis, including molecular identification and secondary metabolites analysis.

**Table 3:** Antimicrobial activity of active isolates against both clinical and ATCC strains.

Isolates	Media	Antimicrobial activity (diameter of inhibition halo in mm ± SD)				
		*Pseudomonas aeruginosa* ATCC 10145	*Escherichia coli* ATCC 3739	Vancomycin-resistant *Enterococcus*	Methicillin-resistant *Staphylococcus aureus*	*Candida albicans*
SAS01	ISP4	–	12 ± 1.52	12 ± 1.00	–	–
	SCA	–	12 ± 1.00	16 ± 0.57	12 ± 1.52	14 ± 0.57
SAS02	ISP4	16 ± 1.52	15 ± 0.00	14 ± 0.057	16 ± 1.73	16 ± 1.15
	SCA	18 ± 0.00	14 ± 2.00	15 ± 2.00	17 ± 1.00	16 ± 0.57
SAS03	ISP4	–	–	18 ± 1.52	13 ± 2.51	–
	SCA	–	–	–	–	18 ± 0.00
SAS04	ISP4	–	14 ± 2.51	–	13 ± 2.00	–
	SCA	–	9 ± 0.57	–	–	13 ± 0.00
SAS05	ISP4	15 ± 1.52	18 ± 0.57	14 ± 1.52	17 ± 0.00	17 ± 1.52
	SCA	18 ± 0.57	8 ± 0.57	14 ± 1.15	12 ± 1.52	9 ± 0.00
SAS06	ISP4	–	20 ± 1.00	22 ± 2.00	22 ± 1.00	–
	SCA	–	20 ± 1.52	13 ± 1.73	19 ± 0.00	22 ± 2.51
SAS08	ISP4	–	–	17 ± 0.57	20 ± 2.08	–
	SCA	–	–	–	–	–
SAS09	ISP4	15 ± 1.52	14 ± 0.00	28 ± 1.00	30 ± 1.15	24 ± 1.15
	SCA	16 ± 0.57	16 ± 0.57	16 ± 2.51	22 ± 1.52	15 ± 0.00
SAS11	ISP4	–	18 ± 2.00	15 ± 0.57	–	–
	SCA	–	–	–	–	–
SAS13	ISP4	16 ± 0.00	18 ± 0.00	17 ± 2.00	18 ± 1.52	26 ± 0.57
	SCA	18 ± 2.00	22 ± 0.57	15 ± 0.57	14 ± 0.57	–
SAS15	ISP4	18 ± 1.00	22 ± 0.00	21 ± 0.57	24 ± 2.51	20 ± 0.57
	SCA	20 ± 2.00	22 ± 3.51	20 ± 0.00	18 ± 2.00	15 ± 1.00
SAS19	ISP4	16 ± 0.57	18 ± 1.00	18 ± 0.00	19 ± 0.57	19 ± 2.00
	SCA	16 ± 1.00	22 ± 2.51	22 ± 2.00	24 ± 0.00	21 ± 0.57

*–, no activity.*

### Molecular Identification and Phylogenetic Analysis

The phylogeny of the selected strains, which showed broad-spectrum antimicrobial activity, was evaluated on the basis of their partial 16S rRNA gene sequences. PCR amplification, sequencing, and processing of 16S rRNA gene fragment yielded final contigs of the size ranging from 1,375 bp to 1,391 bp. The identification of 16S rRNA gene sequence using EzTaxon server revealed that all the four isolates belong to a single genus, *Streptomyces*. **Table 4** summarizes the 16S rRNA gene sequence coverage, accession numbers, and similarity with known strains.

**Table 4:** 16S rRNA gene sequence data in comparison with nearest valid members of *Streptomyces* genus.

Isolate	16S gene data coverage (bp)	Accession number	Maximum similarity (%)	Nearest type strain
SAS02	1,396	KP986569	98.35	*Streptomyces zagrosensis* HM 1154(T) (Mohammadipanah et al., 2014)
SAS09	1,390	KP986570	99.93	*Streptomyces gancidicus* NBRC 15412(T) (Suzuki, 1957)
SAS13	1,391	KP986571	99.86	*Streptomyces tuirus* NBRC 15617(T) (Albert and Malaquias de Querioz, 1963)
SAS15	1,375	KP986572	100.0	*Streptomyces enissocaesilis* NBRC 100763(T) (Gause et al., 1983)

Strain SAS02 showed 93.84–98.35% sequence similarity with the members of genus *Streptomyces*, with the maximum (98.35 %) similarity to *Streptomyces zagrosensis* HM 1154^T^ (Mohammadipanah et al., 2014). Strain SAS09 shared 93.44–98.93% sequence similarity to the members of *Streptomyces*, with the highest similarity to *Streptomyces gancidicus* NBRC 15412^T^ (Suzuki, 1957). Strain SAS13 showed 93.38–99.86% sequence similarity, showing maximum similarity to *Streptomyces tuirus* NBRC 15617^T^ (Albert and Malaquias de Querioz, 1963). Strain SAS15 showed 92.94–100% similarity to the members of *Streptomyces*, with the highest similarity to *Streptomyces enissocaesilis* NRRL B–16365^T^ (Gause et al., 1983). Consequently, SAS02, SAS09, SAS13, and SAS15 were designated as *Streptomyces* sp. SAS02, *Streptomyces* sp. SAS09, *Streptomyces* sp. SAS13, and *Streptomyces enissocaesilis* strain SAS15, respectively.

**FIGURE 4:**
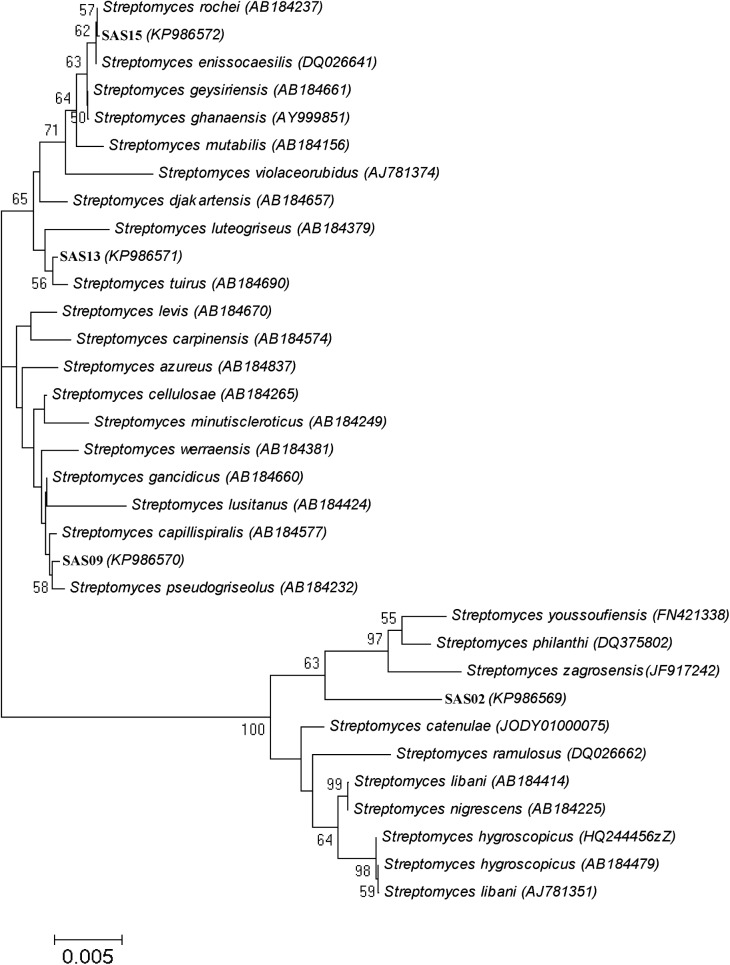
Phylogenetic analysis of selected actinobacterial strains. Neighbor-joining phylogenetic tree showing evolutionary relationships of selected isolates based on 16S rRNA sequence alignments. Organisms represented are the isolates from this study and their closest valid GenBank matches. Numbers at nodes indicate percentages of 1,000 bootstrap resamplings; only values above 50% are given. Bar indicates 0.005 substitutions per nucleotide position.

The neighbor-joining phylogenetic tree analysis segregated SAS02, SAS09, SAS13, and SAS15 into three different clusters (**Figure 4**) representing the genus *Streptomyces*. Strains SAS13 and SAS15 were assigned to the same cluster, while SAS02 and SAS09 were grouped into two separate clusters. Interestingly, strain SAS02 clearly separated from its nearest strain type, suggesting that it may be a novel strain.

### PCR-Based Screening for PKS and NRPS Systems

**FIGURE 5:**
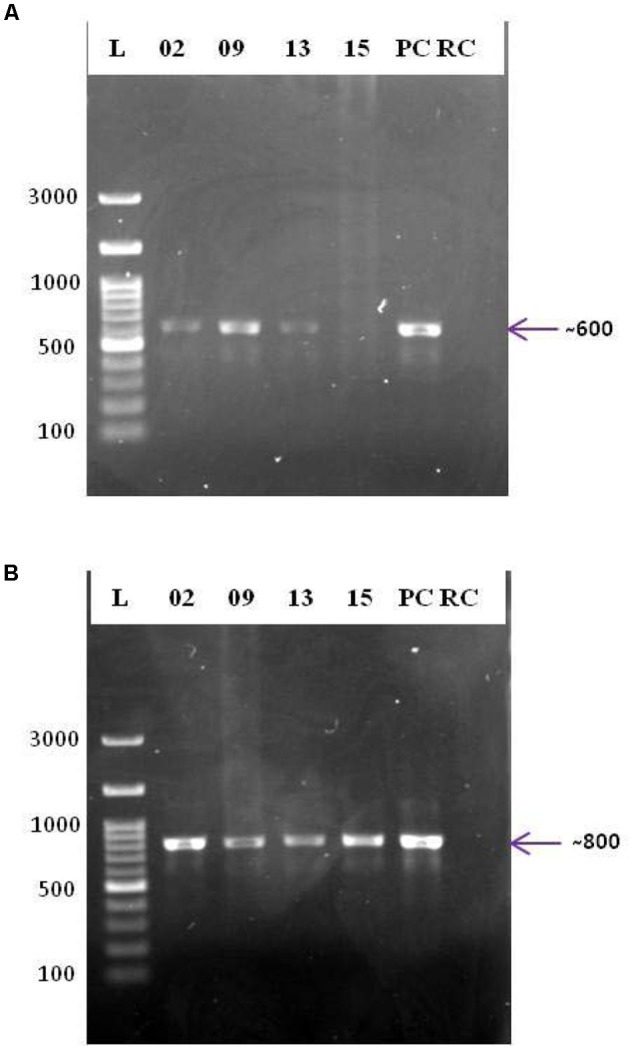
PCR based screening for PKS and NRPS systems. Agarose gels (1%, w/v) showing the amplicons of PCR targeted the ketosynthase domain of type II PKS **(A)** and the adenylation domain of NRPS **(B)**. L: 100 bp DNA ladder; 02, 09, 13, and 15: PCR amplicons of strains SAS02, SAS09, SAS13, and SAS15, respectively; PC, positive control; RC: reaction control.

The biotechnological significance of the isolates was examined using PCR-based screening for type II PKS and NRPS systems (**Figure 5**). Strains SAS02, SAS09, and SAS13 were positive for both type II PKS and NRPS systems, whereas SAS15 was positive only for NRPS system. Presence of type II PKS and NRPS systems proved biosynthetic potential of the selected isolates that showed antimicrobial activity.

### Analysis of the LC/MS Data

Secondary metabolites, present in the crude extracts fractionated with an equal volume of ethyl acetate from the fermentation broths of the selected strains were subjected to high resolution LC-MS-Q-TOF analysis (Chromatograms can be found in Supplementary Figure S1) and database search. The compounds produced by the different strains are summarized in **Table 5**. Notably, the strain SAS02 produced a known microbial bioactive metabolite, related to doxorubicinol, and several putative novel metabolites. Strains SAS09 and SAS13 produced compounds that were related to known compounds like pyrromycin and erythromycin. Similarly, strain SAS15 produced erythromycin derivatives. Further, about 5-12 putatively novel metabolites having molecular features unlike of any other known compounds were also identified. Among them, SAS02 produced 12 putatively novel compounds, followed by SAS15, SAS13, and SAS09 with 9, 6, and 5 novel compounds, respectively (**Table 4**).

**Table 5:** Known and putatively novel secondary metabolites found from the selected isolates in chemical analysis.

Isolate	Known secondary metabolites			Putatively novel secondary metabolites	
	Nos.	Mass (m/z) and related compounds^∗^	Structural class and activity	Nos.	Mass (m/z)
SAS02	1	[M+H]^+^ = 568.1903 Doxorubicinol	Anthracyclines, anticancer activity	12	[M+H]^+^ = 135.0182, 146.032, 174.0224, 129.0087, 254.0471, 265.0616, 489.2129, 265.0617, 449.1854, 489.2149, 526.1938, 537.2806
SAS09	2	[M+H]^+^ = 568.2139 Pyrromycin	Anthracycline glycoside, antibacterial activity	5	[M+H]^+^ = 120.0145, 191.0056, 322.03, 852.3207, 812.3345
		[M+H]^+^ = 790.4954 Erythromycin	Macrolide, antibacterial activity		
SAS13	2	[M+H]^+^ = 568.2149 Pyrromycin	Anthracycline glycoside, antibacterial activity	6	[M+H]^+^ = 129.9969, 147.0222, 550.1804, 731.4479, 376.2484, 825.3443
		[M+H]^+^ = 790.4945 Erythromycin	Macrolide, antibacterial activity		
SAS15	1	[M+H]^+^ = 746.4712 14-hydroxy-6-omethylerythromycin A	Macrolide, antibacterial activity	9	[M+H]^+^ = 210.082, 590.2232, 337.0776, 111.0018, 618.2537, 279.0547, 640.5538, 616.3332, 657.418

*^∗^Compounds were identified by comparing with molecular feature of known compounds available in natural product databases and literatures.*

## Discussion

Actinobacteria from poorly explored habitats are an important source of medically relevant secondary metabolites (Fenical and Jensen, 2006; Bull and Stach, 2007; Monciardini et al., 2014; Singh et al., 2016). India is a country well endowed with natural resources, like large forest area, sea coast, and deserts having diverse climatic conditions; however, they are largely unexplored. In the present study, 21 morphologically distinct Actinobacteria were isolated from arid soil samples collected from a previously unexplored location in the Thar Desert. Although a few studies have recently been resulted in isolation of relatively larger number of isolates from desert ecosystem (Arocha-Garza et al., 2017; Mohamed et al., 2017), this study represents the occurrence of diverse culturable Actinobacteria in a relatively small sampling area (128 m^2^).

Morphological characteristics, notably aerobic growth, chalky and heaped appearance, aerial and substrate mycelia with different colors, and an earthy odor suggested that the isolates are affiliated to genus *Streptomyces*. These characteristics of *Streptomyces* are widely considered for their preliminary identification (Taddei et al., 2006).

The *Streptomyces* are a well-established source of diverse bioactive compounds possessing antimicrobial activity. This agrees well with the current study, which uncovered the antimicrobial activity of 12 different actinobacterial strains. Further, we observed antibacterial activity against MRSA and VRE, implying the need for detailed characterization of the active isolates and their principal components. *Streptomycetes* from deserts have been previously investigated for the production of antimicrobial metabolites. For instance, a new type of ansamycin and 22 members of macrolactones with antibacterial and antitumor activity were isolated from *Streptomyces* species that were isolated from the Atacama Desert soils (Rateb et al., 2011). Similarly, novel Abenquines A–D, which demonstrated inhibitory activity against bacteria and dermatophytic fungi, were purified from a *Streptomyces* strain isolated from the Chilean Atacama Desert (Schulz et al., 2011). The current study further adds to these observations and shows that Thar Desert is a source of actinobacterial strains with a potential for the exploitation of bioactive compounds.

The strains showed potent antimicrobial activity were characterized by 16S rDNA sequence analysis to assign them a particular molecular taxonomic unit. It is interesting to note that three out of four isolates showed dissimilarity to the already reported members of genus *Streptomyces*. Recent studies have raised the 16S rRNA gene sequence similarity threshold to 99% for Actinobacteria (Stach et al., 2003; Guo et al., 2015). Accordingly, strain SAS02 represents a novel species of the genus *Streptomyces*. The taxonomic novelty of SAS02 was further supported by the formation of distinct sub-clade in the neighbor-joining phylogenetic tree.

Microbial natural products with interesting biological activities are mainly synthesized by PKS and NRPS systems (Fischbach and Walsh, 2006). The occurrence of genes that encode such biosynthetic systems in a microorganism strongly indicates their biosynthetic potential (Ayuso-Sacido and Genilloud, 2005). In this report, a PCR-based screening method was adopted for the detection of PKS and NRPS systems in streptomycetes. Interestingly, out of the four strains selected, three were found to be positive for both type II PKS and NRPS systems. The presence of PKS and NRPS systems in the selected strains provides evidence for their biosynthetic potential. Since genome-guided screening of isolates is helpful in the discovery of novel drug leads (Heine et al., 2014; Huang et al., 2016), future studies will focus on the genomes of these isolates.

The application of LC-MS in identifying novel metabolites is a well-recognized methodology (Abdelmohsen et al., 2015; Sun et al., 2015). We could obtain only a qualitative estimate of the abundance of bioactive secondary metabolites in the selected isolates. Janso and Carter (2010) employed this approach to uncover the biosynthetic potential of endophytic Actinobacteria. Similarly, an LC-MS based study by Tiwari et al. (2015) suggested that the culturable Actinobacteria of Indian desert regions are a source of bioactive Actinobacteria. This study further revealed the biosynthetic potential of Actinobacteria inhabiting arid environments.

Among the four selected isolates, known antibacterial compounds related to pyrromycin and erythromycin derivatives were detected from the ethyl acetate extract of SAS09, SAS13, and SAS15. In contrast, known antibacterial compounds were not found from SAS02, which indicates the presence of putatively novel antimicrobial compounds. Moreover, strain SAS02 is a novel strain and supports a fact that antimicrobial activity against the highest impact may come from the novel, undescribed strains present in the unexplored environment (Donadio et al., 2007; Guo et al., 2015; Parrot et al., 2015). Overall, production of 5–12 putatively novel compounds was detected from the selected strains and it is attesting their importance in secondary metabolites production. It is important to note that these compounds were identified from the spent broth of selected isolates by single-solvent (ethyl acetate) extraction, revealing the abundance of secondary metabolites and their complete chemical space may be further studied under a wide range of cultivation conditions, culture media, and secondary metabolites extraction methods. Since the comprehensive exploration of Actinobacteria is indispensable in the discovery of bioactive compounds (Chater and Chandra, 2006; Kersten et al., 2011; Harrison and Studholme, 2014), further efforts are necessary for the structural characterization of the metabolites produced by SAS02 under different laboratory conditions, and whole genome sequencing is required to explore its complete potential.

## Conclusion

The research on the microbial natural product is expected to move ahead in a new direction, aided by the innovations in Next Generation Sequencing and spectroscopic methods. This study reports an investigation of the biosynthetic potential of Actinobacteria from the soil samples collected from the semi-arid region of India. The relatively small number of isolates screened here demonstrates their novelty, significance, and biosynthetic potential. Actinobacteria thus identified seem to be a promising source of new and interesting natural products that will be further explored for their biotechnological applications.

## Author Contributions

The work was conceived and designed by SJ, PJ, EM, and GS. Experiments were done by MM, KS, and TT. Data analysis and verification were done by RA, NS, MM, and KS. The manuscript was drafted by KS, MM, and PJ. The manuscript was approved by all the authors.

## Conflict of Interest Statement

The authors declare that the research was conducted in the absence of any commercial or financial relationships that could be construed as a potential conflict of interest.
