# Supplementary material for: Biosynthetic Potential of Bioactive Streptomycetes Isolated From Arid Region of the Thar Desert, Rajasthan (India)
Source: Front Microbiol. 2018 Apr 18;9:687. doi: 10.3389/fmicb.2018.00687 (PMC5915549; doi:10.3389/fmicb.2018.00687)
Supplement: Supplementary file 1 [file Data_Sheet_1.pdf]

Supplementary Materials

Biosynthetic potential of bioactive streptomycetes isolated from arid region of the Thar Desert, Rajesthan (India)

Meeta Masand[1‡], Kunjukrishnan Kamalakshi Sivakala[2‡] Ekta Menghani[3], Thangathurai Thinesh[4], Rangasamy Anandham[5], Gaurav Sharma[1], Natesan Sivakumar[2], Solomon RD Jebakumar[2ǂ], Polpass Arul Jose[2,*†]

[1]School of Life sciences, Suresh Gyan Vihar University, Jaipur, India

[2]Department of Molecular Microbiology, School of Biotechnology, Madurai Kamaraj University, India

[3]Department of Biotechnology, School of Science, JECRC University, Jaipur, India

[4]Department of Microbiology, School of Life-sciences, Pondicherry University, Puducherry, India

[5]Department of Agricultural Microbiology, Agricultural College and Research Institute, Tamil Nadu Agricultural University, Madurai, India

Running title: Biosynthetic potential of bioactive streptomycetes

[‡]These authors have contributed equally to this work.

[ǂ]Deceased

*Correspondence

Dr. Polpass Arul Jose

Email: arulmku@gmail.com

[†]Current Address: Marine Biotechnology and Ecology Division, Central Salt and Marine Chemicals Research Institute, Bhavnagar, Gujarat, India.

Figure S1: HR LC-MS chromatogram of ethylacetate extract from strains, SAS02 (A), SAS09 (B), SAS13 (C) and SAS15 (D)

A)

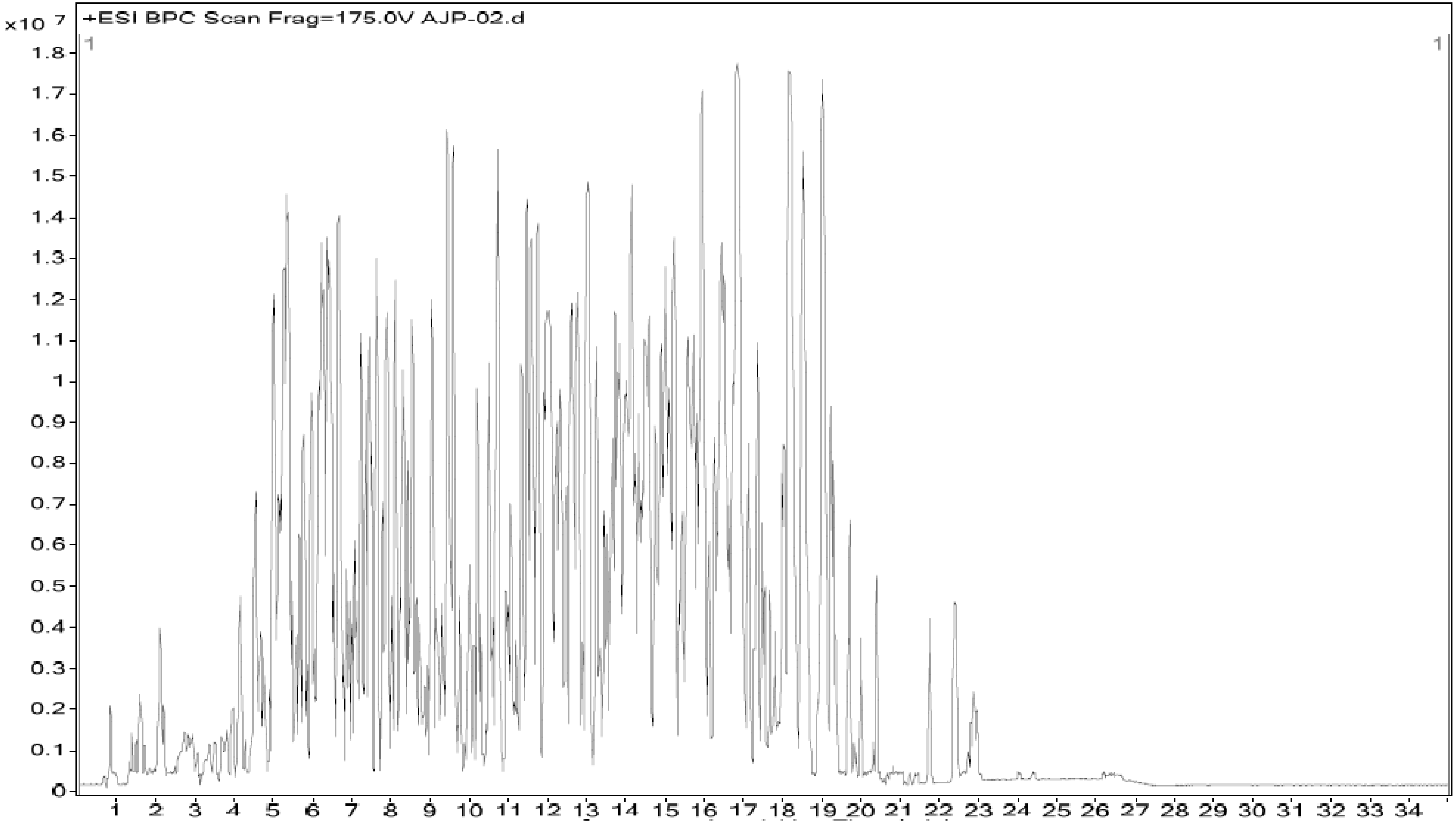

B)

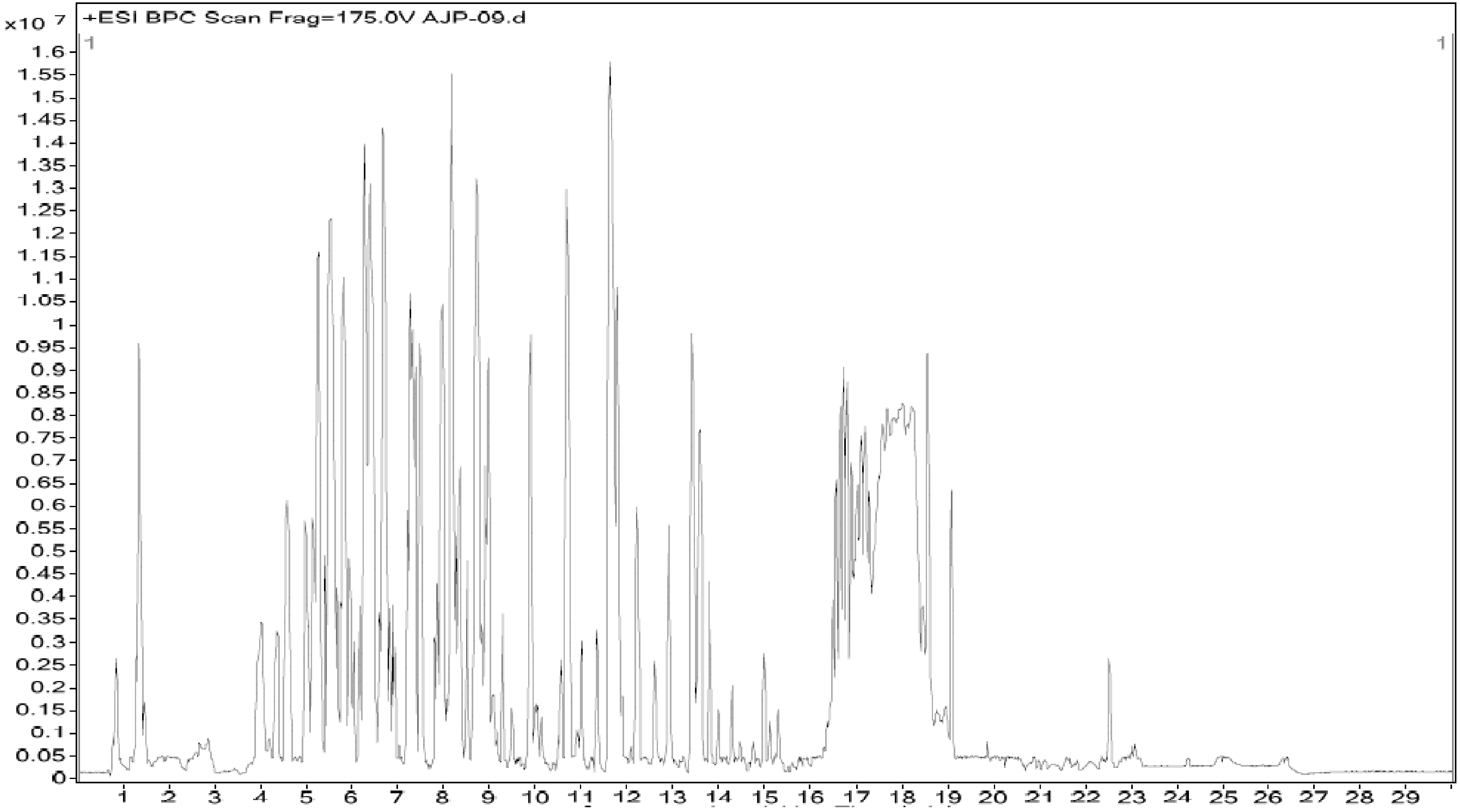
+ESI BPC Scan Frag=175.0V AJP-09.d
x10 7
1.6
1.55
1.5
1.45
1.4
1.35
1.3
1.25
1.2
1.15
1.1
1.05
1
0.95
0.9
0.85
0.8
0.75
0.7
0.65
0.6
0.55
0.5
0.45
0.4
0.35
0.3
0.25
0.2
0.15
0.1
0.05
0
1 2 3 4 5 6 7 8 9 10 11 12 13 14 15 16 17 18 19 20 21 22 23 24 25 26 27 28 29

c)

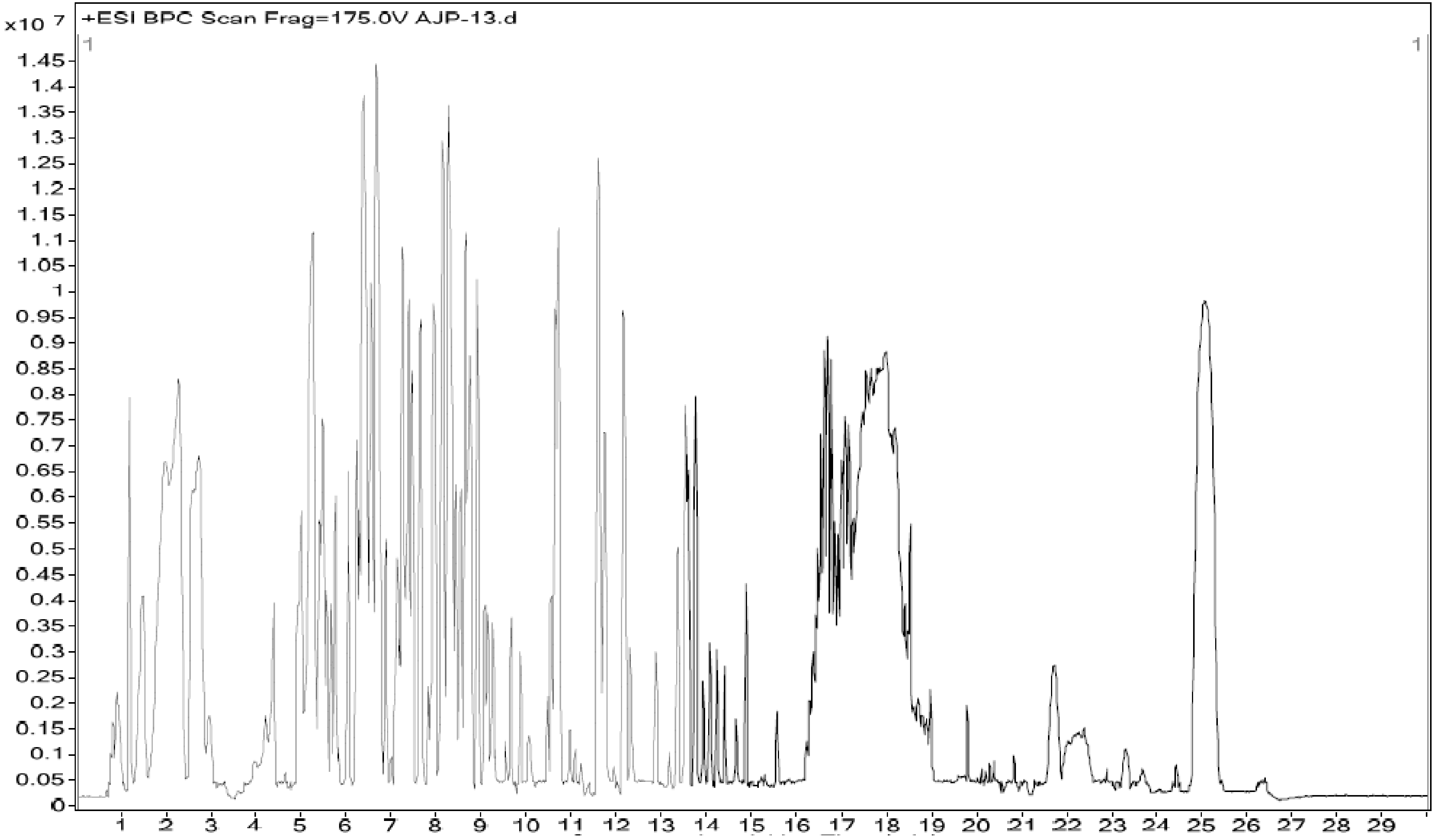

+ESI BPC Scan Frag=175.0V AJP-13.d
x10 7
1.45
1.4
1.35
1.3
1.25
1.2
1.15
1.1
1.05
1
0.95
0.9
0.85
0.8
0.75
0.7
0.65
0.6
0.55
0.5
0.45
0.4
0.35
0.3
0.25
0.2
0.15
0.1
0.05
0
1 2 3 4 5 6 7 8 9 10 11 12 13 14 15 16 17 18 19 20 21 22 23 24 25 26 27 28 29

D)

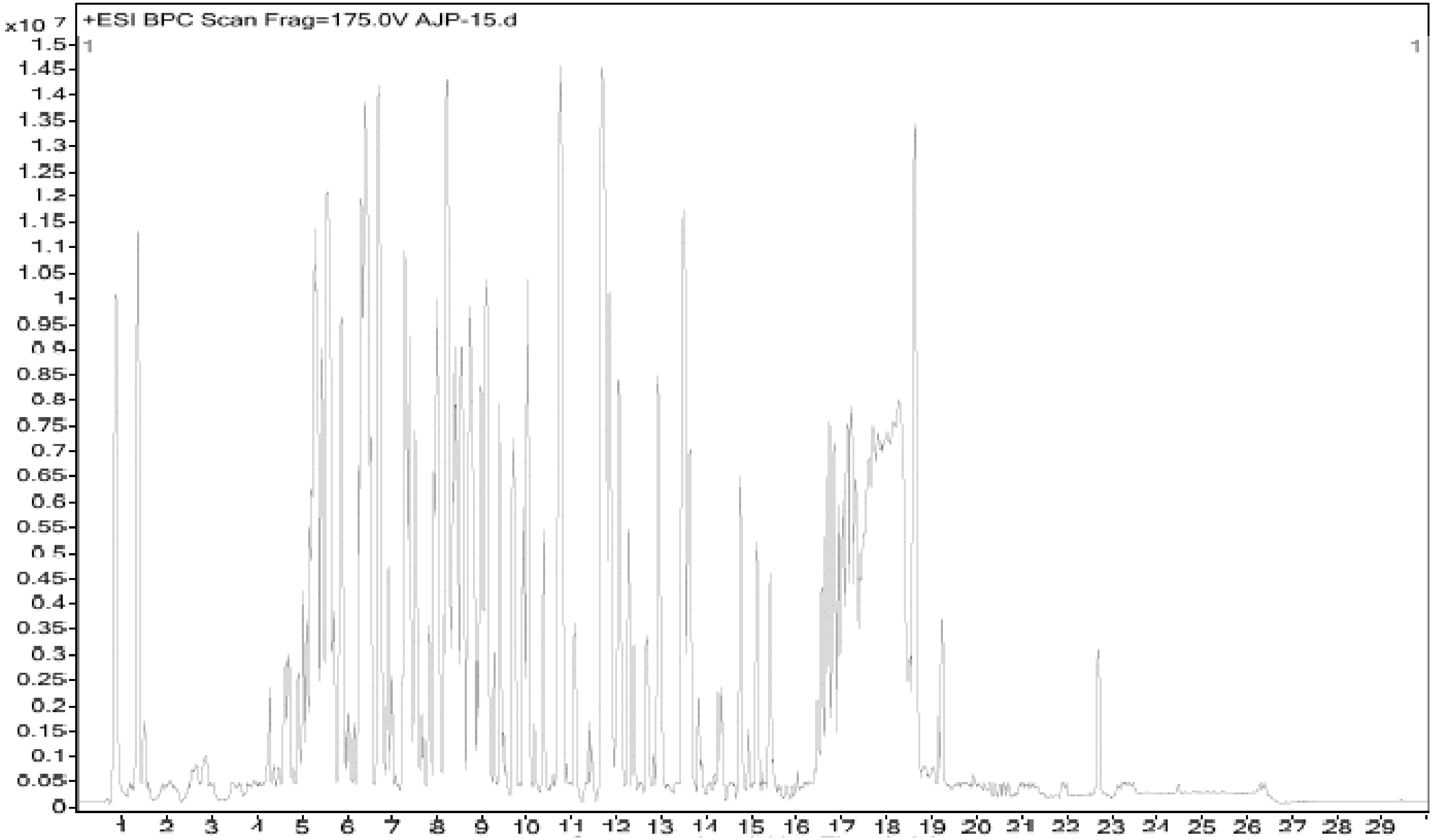
+ESI BPC Scan Frag=175.0V AJP-15.d
x10 7
1.5
1.45
1.4
1.35
1.3
1.25
1.2
1.15
1.1
1.05
1
0.95
0.9
0.85
0.8
0.75
0.7
0.65
0.6
0.55
0.5
0.45
0.4
0.35
0.3
0.25
0.2
0.15
0.1
0.05
0
1 2 3 4 5 6 7 8 9 10 11 12 13 14 15 16 17 18 19 20 21 22 23 24 25 26 27 28 29
